# Extracorporeal photopheresis reduces inflammation and joint damage in a rheumatoid arthritis murine model

**DOI:** 10.1186/s12967-024-05105-x

**Published:** 2024-03-25

**Authors:** Yuwei Lin, Zhanrui Cheng, Yan Zhong, Yinting Zhao, Guifen Xiang, Ling Li, Li Tian, Zhong Liu

**Affiliations:** 1 School of Public Health, Anhui Medical University, Hefei, 230032 China; 2 Institute of Blood Transfusion, Chinese Academy of Medical Sciences and Peking Union Medical College, Chengdu, 610052 China; 3 Key Laboratory of Transfusion Adverse Reactions, Chinese Academy of Medical Sciences, Chengdu, 610052 China; 4 Department of Blood Transfusion, The Third People’S Hospital of Chengdu, College of Medicine, Southwest Jiaotong University, Chengdu, 610031 China; 5 School of Population Medicine and Public Health, Chinese Academy of Medical Sciences & Peking Union Medical College, Beijing, 100730 China

**Keywords:** Extracorporeal photopheresis, Collagen-induced arthritis, Rheumatoid arthritis, Cellular therapy

## Abstract

**Background:**

Rheumatoid arthritis (RA) is an autoimmune disease characterized by inflammatory reactions and tissue damage in the joints. Long-term drug use in clinical practice is often accompanied by adverse reactions. Extracorporeal photopheresis (ECP) is an immunomodulatory therapy with few side effects, offering a potential and safe therapeutic alternative for RA through the induction of immune tolerance. This study aimed to investigate the therapeutic effects of ECP on RA using a collagen-induced arthritis (CIA) murine model, as well as to explore its immunomodulatory effects in vivo. Additionally, particular attention was given to the significant role of monocytes during the ECP process.

**Methods:**

A murine model of rheumatoid arthritis was established by administering two injections of bovine type II collagen to DBA/1J mice. ECP, ECP-MD (mononuclear cells were depleted during the ECP), MTX, and PBS treatment were applied to the CIA mice. During the treatment process, clinical scores and body weight changes of CIA mice were closely monitored. After six treatment sessions, micro-CT images of the hind paws from live mice were captured. Ankle joints and paws of the mice were collected and processed for histological evaluation. Spleen samples were collected to measure the Th17/Treg cells ratio, and serum samples were collected to assess cytokine and anti-type II collagen IgG levels. Monocytes and dendritic cells populations before and after ECP in vitro were detected by flow cytometry.

**Result:**

ECP therapy significantly attenuated the progression of CIA, alleviated the severity of clinical symptoms in CIA mice and effectively suppressed synovial hyperplasia, inflammation, and cartilage damage. There was an expansion in the percentage of CD3 + CD4 + CD25 + FoxP3 + Tregs and a decrease in CD3 + CD4 + IL17A + Th17 cells in vivo. Furthermore, ECP reduced the serum levels of pro-inflammatory cytokines IL-6 (53.47 ± 7.074 pg/mL vs 5.142 ± 1.779 pg/mL, *P* < 0.05) and IL-17A (3.077 ± 0.401 pg/mL vs 0.238 ± 0.082 pg/mlL, *P* < 0.0001) compared with PBS. Interestingly, the depletion of monocytes during the ECP process did not lead to any improvement in clinical symptoms or histological scores in CIA mice. Moreover, the imbalance in the Th17/Treg cells ratio became even more pronounced, accompanied by an augmented secretion of pro-inflammatory cytokines IL-6 and IL-17A. In vitro, compared with cells without ECP treatment, the proportion of CD11b + cells were significantly reduced (P < 0.01), the proportion of CD11c + cells were significantly elevated (P < 0.001) 24 h after ECP treatment. Additionally, the expression of MHC II (P < 0.0001), CD80 (P < 0.01), and CD86 (P < 0.001) was downregulated in CD11c + cells 24 h after ECP treatment.

**Conclusion:**

Our study demonstrates that ECP exhibits a therapeutic effect comparable to conventional therapy in CIA mice, and the protective mechanisms of ECP against RA involve Th17/Treg cells ratio, which result in decreased IL-6 and IL-17A. Notably, monocytes derived from CIA mice are an indispensable part to the efficacy of ECP treatment, and the proportion of monocytes decreased and the proportion of tolerogenic dendritic cells increased after ECP treatment in vitro.

**Supplementary Information:**

The online version contains supplementary material available at 10.1186/s12967-024-05105-x.

## Introduction

Rheumatoid arthritis (RA) is an autoimmune disease that can occur at any age. The immune system misrecognizes connective tissues in the joints as foreign bodies or pathogens and launches immune attacks, leading to inflammatory reactions and tissue damage in these joints. Eventually, irreversible deformities and loss of function develop, complicated by pulmonary diseases, cardiovascular diseases, malignant tumors, and depression [1–4]. Unfortunately, there is no definitive cure for RA. The main drugs used for RA treatment are disease-modifying antirheumatic drugs (DMARDs), non-steroidal anti-inflammatory drugs (NSAIDs), glucocorticoids, herbal preparations, and biological agents [5]. However, the remission rate after treatment with these drugs is low [6], and long-term drug use is accompanied by adverse reactions such as metabolic disorders and infections [7]. Therefore, there is a pressing demand for the innovation of novel therapeutic strategies for RA that are both more efficacious and have a reduced toxicity profile. The exact pathogenesis of RA remains unclear, research suggests that an imbalance in the T helper 17 (Th17) cells / regulatory T cells (Tregs) may play a crucial role in the progression of RA [8]. In light of this, research focusing on strategies to modulate the balance between Tregs and Th17 cells holds significant value for the treatment of RA.

Extracorporeal photopheresis (ECP) is a transfusion therapy that utilizes the biological effects generated by photosensitizers under photodynamic action. The ECP procedure primarily involves the collection of the leukocyte layer from patient, addition of the photosensitizer 8-methoxypsoralen (8-MOP) solution, irradiation with ultraviolet A (UVA), and reinfusion into the patient [9]. ECP has demonstrated effective and safe therapeutic outcomes in the clinical management of various T cell-mediated disorders [10, 11]. For instance, in patients with graft-versus-host disease (GVHD) or solid organ transplant rejection, the prophylactic or therapeutic use of ECP has shown specific and long-term immunosuppressive effects [12–15]. Moreover, prospective studies conducted on ECP treatment for other autoimmune disease like systemic sclerosis, Crohn’s disease, and type 1 diabetes, pemphigus vulgaris and other autoimmune diseases, have also revealed promising results [16–18]. Given the case report, there are also some RA patients benefited from the ECP therapy, the clinical symptoms were relieved and controlled [19–21]. Although ECP has shown favorable results in the treatment of these diseases, the mechanisms underlying its induction of immune tolerance remain unclear. Existing studies suggest that ECP may induce monocyte differentiation into tolerogenic dendritic cells, stimulate an increase in Tregs, and release anti-inflammatory cytokines in patients with GVHD [22]. However, there is currently no robust evidence to support whether similar mechanisms can lead to therapeutic effects in patients with RA.

The lack of support from preclinical studies and the limited understanding of the mechanisms underlying the action of ECP, have hindered its widespread application in RA. In this study, we utilize a CIA murine model to investigate the therapeutic effects of ECP on RA and its regulation both in Treg/Th17 immune cells and related cytokines, as well as whether monocytes play a role during the ECP treatment process.

## Materials and methods

### Mice

Male DBA/1J mice aged 7–8 weeks were purchased from Chengdu Ensiweier Biological Technology co., Ltd (certificate number: SCXK 2019-0004). The animals were supplied with sterile water and food and kept in a specific-pathogen-free (SPF) facility, where the temperature (22–24℃), humidity (45–60%) and a 12/12 h light–dark cycle was maintained. The animals underwent the experiment after a 1 week acclimation period. All protocols were reviewed and approved by the Ethics Review Committee of Institute of Blood Transfusion, Chinese Academy of Medical Sciences (NO: 2021051).

### Reagents

The following reagents were utilized in our research: complete freund’s adjuvant (CFA), incomplete freund’s adjuvant (IFA), bovine type II collagen and mouse anti type II collagen IgG antibody assay kit were purchased from Chondrex. Mouse mononuclear separation kit were purchased from Solarbio. Methotrexate (MTX), tween-80 and polyethylene glycol 300 were purchased from MedChemExpress. Mouse Th1/Th2/Th17 CBA kit and fluorochrome-coupled antibodies were purchased from B&D Biosciences.

### Collagen-induced arthritis (CIA) murine model

For the primary immunization, mice were subcutaneous inject in the tail with the emulsion of type II bovine collagen and CFA (100 µg collagen/mouse) on day 0. Insert the needle in 2 cm from the tail base until the tip was 0.5 cm from the base. Administer a booster injection consisting of a type II bovine collagen and IFA emulsion on day 21. The booster injection was performed at a different location from the initial injection, further away from the base of the tail. Arthritis symptoms manifested between days 29 and 37 following the initial immunization. The sterile water and food were placed in their cage after day 29 because of the decreased mobility.

### Mice weight and arthritic score

Mice were closely monitored, weighted and scored every other day after the booster collagen injection. Qualitative scoring system were used to assess severity of each paw inflammation as follow: 0 = Normal, absence of inflammation; 1 = Mild inflammation, characterized by distinct redness and swelling of either the ankle or wrist, or noticeable redness and swelling confined to individual fingers or toes, regardless of the number affected; 2 = Moderate inflammation, with evident redness and swelling encompassing the ankle or wrist; 3 = Severe inflammation, encompassing redness and swelling of the entire paw, including fingers or toes; 4 = Maximum level of inflammation, with extensive involvement of multiple joints. The total arthritic score for each mouse was calculated by summing the individual scores for all four paws, with the highest possible total score being 16. Scoring was performed by three independent investigators. Experimental treatment was initiated once the mean arthritic score reached 5.

### Experimental intervention by ECP and MTX

**Fig. 1:**
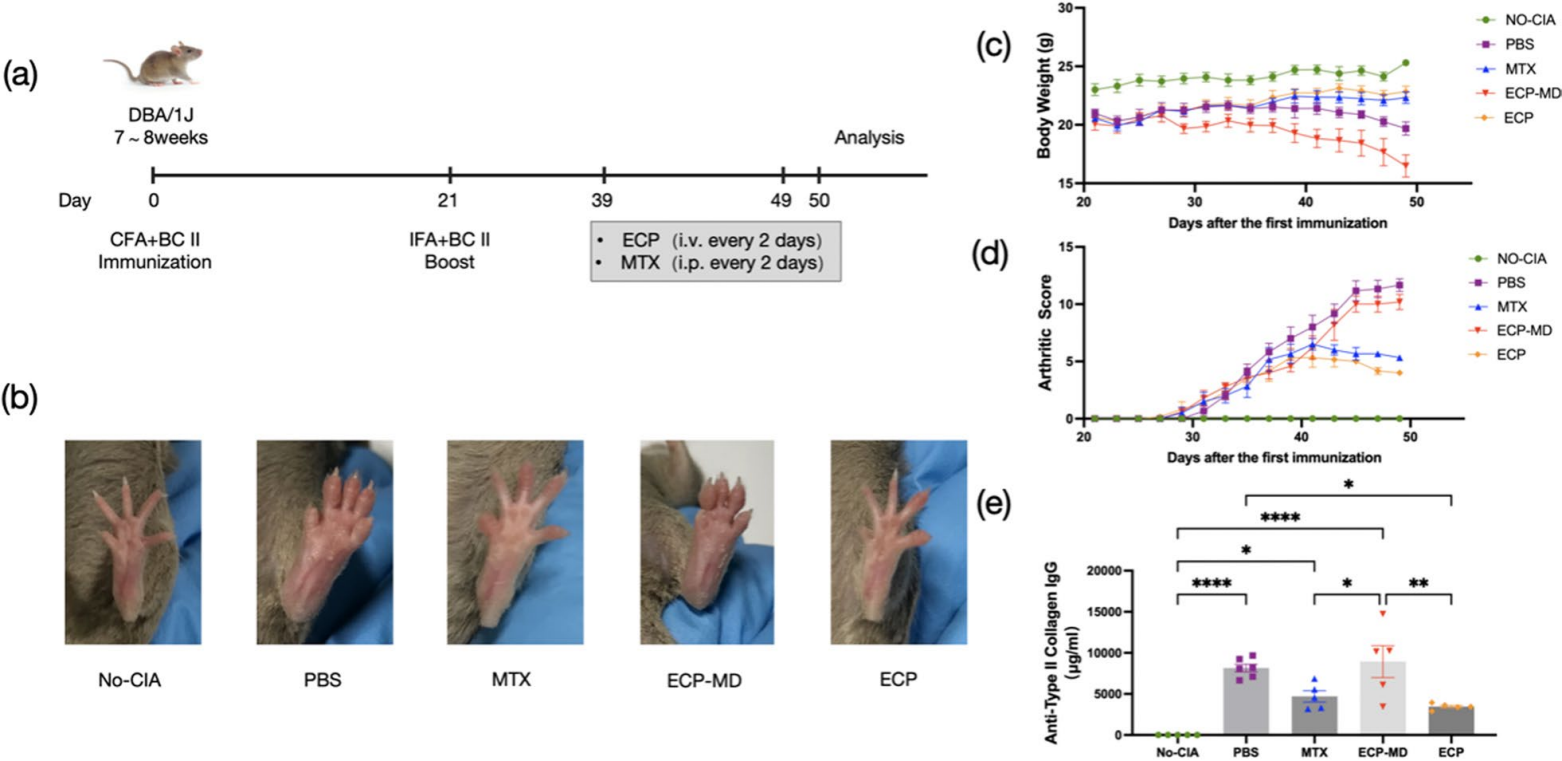
ECP treatment mitigates the clinical manifestations of collagen-induced arthritis (CIA) mice. **a** The schematic outlines the protocol for establishing the CIA murine model and the subsequent experimental procedures. **b** Hind paw images exemplify the reduction of swelling and ankylosis in CIA mice, with the images captured on Day 50 (**c**) Body weights of mice were recorded every 2 days following booster immunization. **d** Arthritic scores of mice were recorded every 2 days after booster immunization. **e** Serum anti-type II collagen IgG concentrations were measured using Enzyme-Linked Immunosorbent Assay (ELISA). Data are shown as mean ± SEM. *P ≤ 0.05, **P ≤ 0.01, ***P ≤ 0.001, ****P ≤ 0.0001

To investigate the effect of ECP in CIA murine model, we conducted the experiment as shown in Fig. 1a. Following the initial injection (Day 0), there was a general trend of body weight loss among CIA mice, leading to the exclusion of any mouse with body weight falling below 18 g. Subsequently, on the day subsequent to the administration of the second booster injection, CIA mice were completely randomized into experimental groups. At the time of randomization, the absence of clinical signs of arthritis, such as visible paw swelling, was noted, which served to minimize biological variability across the groups. CIA mice were divided into four groups (n = 6): PBS, MTX, ECP-MD, and ECP groups, while the No-CIA group (n = 6) were consisted of healthy mice. In the PBS group, mice were given sterile 1×PBS, and in the No-CIA group, the mice remained healthy. MTX was selected as the positive control, and mice were intraperitoneal injection with a dosage of 1.5 mg/kg.

Spleens of donor CIA mice were isolate and venous blood was collected from the inferior vena cava after anesthetizing with 1.25% Tribromoethanol. The samples were subjected to density gradient separation by Mouse Mononuclear Cell Isolation Kit according to the manufacturer in order to obtain mononuclear cells from both mouse spleen and blood. After the washed cells (1*10^7^ cells/ml) were incubated with 200 ng/ml 8-MOP for 15 min in the dark at 37 ℃, followed by UVA irradiation (365 nm, 2J/cm^2^). The cells were then washed twice with 1×PBS. Subsequently, the CIA mice were intravenously injected with mononuclear cells treated with 8-MOP/UVA every other day in this group, designated as ECP. To determine whether monocytes play an important role in ECP, another group named ECP-MD was established. Mononuclear cells obtained from donor mice were depleted of monocytes using the EasySep^™^ Mouse CD11b Positive Selection Kit before undergoing 8-MOP/UVA.

### Histological analysis

On day 50, the mice were euthanized, and their ankle joints were collected. After fixation in 4% paraformaldehyde in phosphate buffer for 24 h, the joints and paws were subjected to a 62 day decalcification process and subsequently embedded in paraffin. Tissue Sects. (3 μm thick) were utilized for both hematoxylin and eosin (H&E) staining and safranin-O staining. Histologic examination was conducted using a microscope (Eclipse Ti, Nikon) and digital pathology scanner (3D-HISTECH). Synovial inflammation and hyperplasia were assessed by H&E staining based on the following criteria [23, 24]: 0 = no inflammation and synoviocyte hyperplasia; 1 = slight synoviocyte hyperplasia, defined as a synoviocyte thickness of less than 4 layers, or minimal scattered inflammatory cell infiltration; 2 = slight synoviocyte hyperplasia and limited scattered inflammatory cell infiltration; 3 = moderate synovial cell proliferation, with a synoviocyte thickness exceeding 4 layers and associated with reduced joint space, coupled with moderate inflammatory cell infiltration, denoted by the aggregation of more than 2 inflammatory cells; 4 = severe synoviocyte hyperplasia, evidenced by the obliteration of the joint space and extensive inflammatory cell infiltration extending into the synovial space. Cartilage damage was evaluated by safranin-O staining based on the following criteria: 0 = smooth joint surface without any erosion; 1 = trace bone erosion, characterized by a single, small, and shallow erosive point; 2 = slight bone erosion, visible as 2–4 erosive points within a limited area and characterized by a dull hue; 3 = moderate bone erosion accompanied by ulceration of the articular surface, with more than 5 erosive points reaching the deep layers of the cartilage; 4 = severe bone erosion with complete erosion of the cortical bone and exposure of the subchondral bone.

### Micro-computed tomography (micro-CT)

Micro-CT (Hiscan XMD Micro-CT System; Hiscan) was used to scan the hind paws images of mice from all five groups on day 49. Mice were anesthetized with isoflurane gas and scanned on a tube at the X-ray source of 80 kV, 100 µA, the resolution ratio of 25 µm.

### Enzyme-linked immunosorbent assay (ELISA)

High IgG autoantibody levels to mouse type II collagen are important for arthritis. Mouse anti-type II collagen antibodies (IgG) were detected by ELISA kits according to the manufacturer’s instructions. The dilution of mice sera is 1:10,000. Absorbance was read at 450 nm, and 630 nm filter was used as a reference using a microplate reader (SpectraMax i3x; Molecular Devices).

### Cytokine assay

Serum samples were collected from 5 groups of mice, and the concentrations of cytokines IL-2, IL-4, IL-6, IL-10, IL-17A, interferon-γ and tumor necrosis factor were detected by a flow cytometric bead array kit, adhering to the supplier’s protocol. The assays were conducted using a flow cytometer (FACS Canto, BD Biosciences).

### Determination of tregs and Th17 cells populations

To evaluate subsets of splenic lymphocytes, flow cytometry was employed. Splenic cells were isolated from anesthetized mice, and erythrocytes were removed using a lysing buffer. Afterwards, the lymphocytes underwent a 6 h incubation with a leukocyte activation cocktail in a humidified CO_2_ incubator at 37 °C. Post-activation, cells were labeled with a fixable viability stain and mouse Fc blocking reagent, followed by incubation on ice. Subsequently, cells were stained with monoclonal antibodies targeting mouse CD3, CD4, CD25, IL-17A, and Foxp3 as per the supplier’s recommendations. After a final wash, the stained samples were run on the flow cytometer (FACS Canto, BD Biosciences).

### Determination of monocytes and dendritic cells populations after experimental ECP in vitro

Mononuclear cells were isolated from CIA mice as described above and treated with 8-MOP (20 ng/ml) and UVA (2J/cm^2^). The treated cells were then collected, washed, and cultured in RPMI complete medium at 37 ℃, 5% CO_2_. After 24 h, the cells were harvested for flow cytometry. Cells that did not undergo ECP (No-ECP) and cells treated with ECP for 24 h (ECP-24 h) were stained with monoclonal antibodies targeting mouse CD11b, CD11c, MHC Class II (I-A/I-E), CD80, and CD86 using standard protocols. After a final wash, the stained samples were run on the flow cytometer (FACS Canto, BD Biosciences) for analysis.

### Statistics

Data processing and statistical analysis were performed using GraphPad Prism version 5.0 software. Comparisons between two groups were conducted using Student’s t-tests. For comparisons among multiple groups, normality was first assessed via the Shapiro–Wilk test. If the data were found to be normally distributed and of equal variance, one-way or two-way analysis of variance (ANOVA) was employed, followed by Tukey’s multiple comparisons test for further analysis. If the data did not meet the assumptions of normality or equal variance, the non-parametric Kruskal–Wallis test was utilized. All data are represented as mean ± SEM. The significance level was set at 0.05.

## Result

### ECP treatment mitigates the clinical manifestations of CIA mice

Mice received twice injection of the emulsion of type II bovine collagen on day 0 and 21 to establish the CIA model (Fig. 1a). Arthritis severity and body weight were assessed every 2 days after booster injections. Paws of CIA mice in PBS group exhibited severe inflammatory responses as indicated by paw redness and swelling compared to No-CIA mice after day 29. When the arthritis scores were generally above 5, the CIA mice were subsequently treated with PBS/MTX/ECP once every 2 days (Fig. 1a). The inflammatory responses were markedly reduced in the ECP and MTX-treated CIA mice (Fig. 1b). The body weight of mice in the No-CIA, ECP, and MTX groups exhibited a steady increase. In comparison to the No-CIA mice, the CIA mice experienced significant body weight loss after the initial immunization, but the treatment of ECP and MTX improve this condition. Furthermore, starting from day 39, the body weight of the ECP mice was significantly higher than that of the PBS group (*P* < 0.05; Fig. 1c). From day 45 onwards, the MTX and ECP groups demonstrated significantly lower arthritis scores in comparison to the PBS group (*P* < 0.05; Fig. 1d). Besides, the effect of ECP on the reduction of arthritis score was slightly better than that of MTX, although the difference was not significant (Fig. 1d).

Type II collagen antibody (anti-CII) plays an important role in CIA lesions and is closely related to arthritis severity, so we used ELISA to measure serum anti-CII content in all mice at the end of treatment. Compare to PBS group, ECP mice showed an obvious decrease in anti-type II collagen IgG (*P* < 0.05; Fig. 1e). Taken together, these findings demonstrate the positive therapeutic effects of ECP in the murine model of CIA, showing a comparable effect to conventional MTX therapy.

### ECP treatment attenuates pathological symptoms in CIA mice

**Fig. 2:**
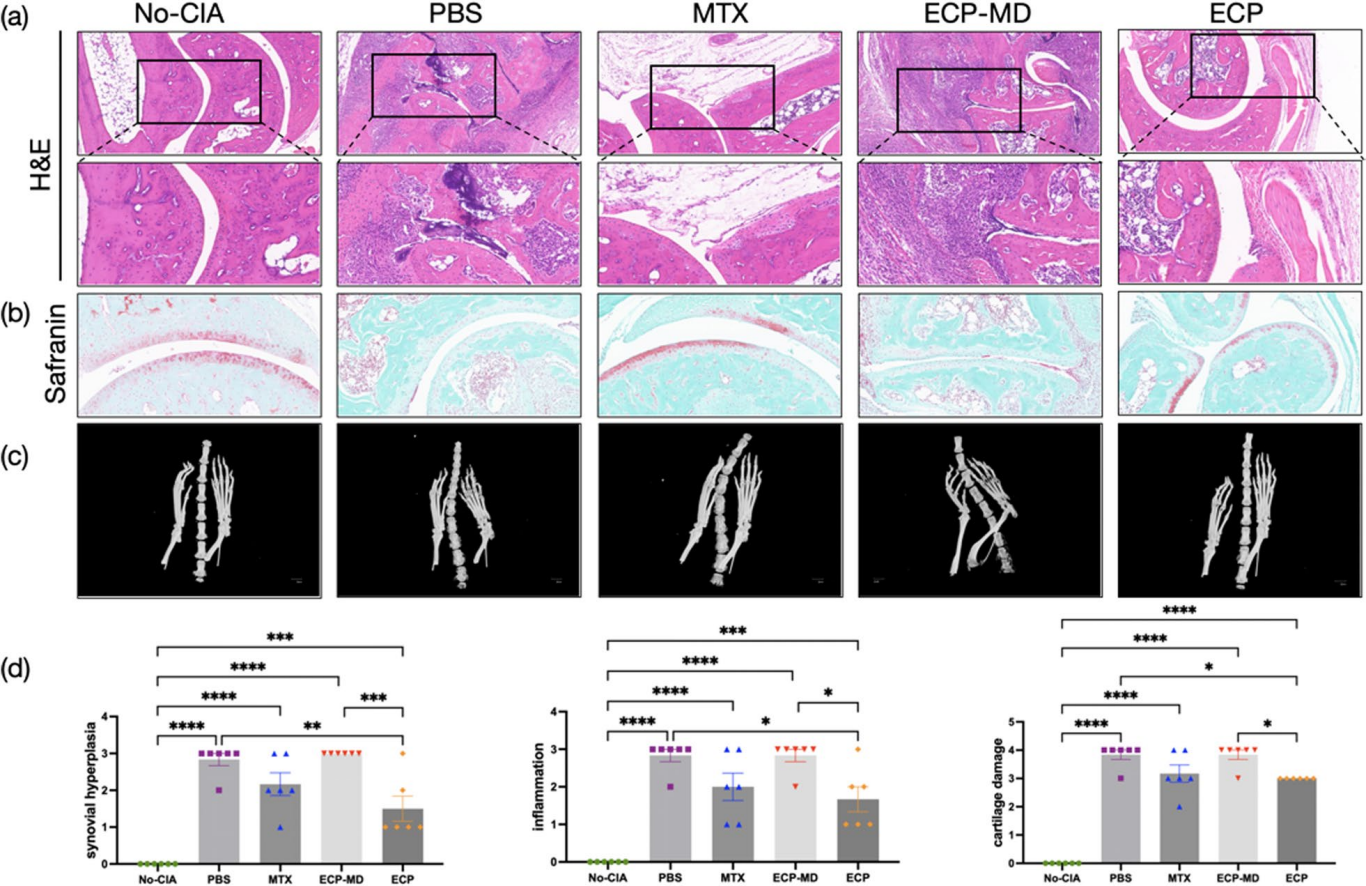
ECP treatment attenuates pathological symptoms in CIA mice. **a** Ankle joints tissue Sects. (3 μm) were utilized for hematoxylin and eosin (H&E) staining, with representative images from each experimental group. **b** Representative images of ankle joints tissue sections were utilized for Safranin-O staining. **c** On day 49, images of live mice paws were captured and used to create 3D reconstructions via micro-computed tomography (micro-CT). **d** Inflammation and synovial hyperplasia were assessed by H&E staining and cartilage damage was assessed by safranin-O staining with a scoring range of 0 to 4. Data are shown as mean ± SEM. n = 5–6 for each group. *P ≤ 0.05, **P ≤ 0.01, ***P ≤ 0.001, ****P ≤ 0.0001

To assess the efficacy of ECP in mitigating arthritic inflammation, hyperplasia and cartilage degradation in CIA mice, histological sections of ankle joints from all five experimental groups were prepared. Histopathological analysis of the PBS mice revealed hallmark pathological alterations indicative of arthritis, including excessive synovial cell proliferation, pervasive infiltration by inflammatory cells, extensive cartilage and osseous tissue erosion, fibrosis, and a progressive narrowing of the articular space. In contrast, both MTX and ECP treatment significantly attenuated these deleterious changes in the ankle joints of CIA mice, demonstrating a clear therapeutic advantage over the PBS-treated controls (Fig. 2a, b). Furthermore, the ECP group exhibited significantly lower scores for synovial hyperplasia, inflammation, and cartilage damage compared to the PBS group (*P* < 0.05; Fig. 2d). Micro-CT scans of the hind paws from live mice exhibited articular destruction in the PBS mice, which were markedly alleviated in both the MTX and ECP groups (Fig. 2c). These results suggest that ECP treatment attenuates the ankle bone and cartilage tissue damage in CIA mice, on par with the conventional therapy MTX.

### ECP treatment modulates the polarization between Tregs and Th17 cells in CIA mice

**Fig. 3:**
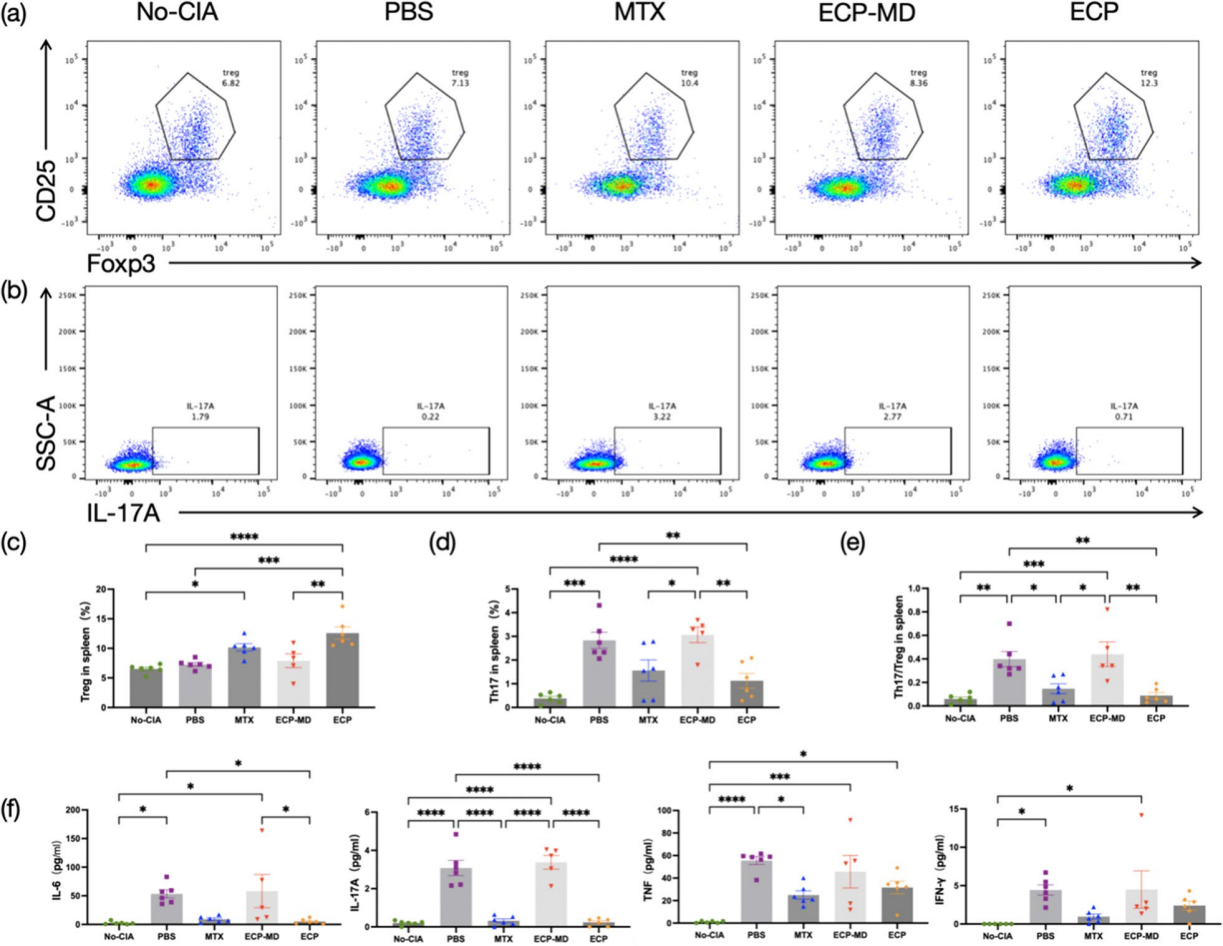
ECP treatment modulates the polarization between Tregs and Th17 cells in CIA mice. **a** The flow cytometry dot plots depict Tregs in No-CIA, PBS, MTX, ECP-MD and ECP-treated mice. **b** The flow cytometry dot plots depict Th17 cells across the same groups. **c**–**e** The splenic proportions of Tregs and Th17 cells were determined and are represented as individual data points. **f** Serum concentrations of cytokines IL-6, IL-17A, tumor necrosis factor and interferon-γ were measured for all groups. Data are shown as mean ± SEM. n = 5–6 for each group. *P ≤ 0.05, **P ≤ 0.01, ***P ≤ 0.001, ****P ≤ 0.0001

Considering the potential of Tregs as a promising therapeutic strategy for human rheumatoid arthritis, and the association of Th17 cells with arthritis initiation and progression, this study aimed to determine if the protective effects of ECP on RA were associated with in vivo modulation of Tregs and Th17 cells populations (Fig. 3a, b). The data demonstrated a substantial elevation of CD3 + CD4 + IL17A + Th17 cells in the PBS-treated mice relative to the No-CIA group. Conversely, these cells were significantly lower in ECP mice compared to PBS mice (P < 0.01; Fig. 3c). Moreover, ECP therapy elicited an expansion of CD3 + CD4 + CD25 + Foxp3 + Tregs in the spleens of ECP-treated mice, in comparison to those treated with PBS (*P* < 0.001; Fig. 3d). The Th17/Treg cells ratio was significantly attenuated in both ECP and MTX-treated mice when juxtaposed with PBS-treated mice (*P* < 0.01 & *P* < 0.05; Fig. 3e). These results suggest that ECP treatment is capable of steering the immune balance by favoring Tregs expansion and reducing Th17 cells proportions in CIA mice, potentially even offering more pronounced therapeutic benefits than conventional MTX therapy.

### ECP treatment reduces inflammation in CIA mice

The modulation of immune cell polarization induced changes in inflammatory cytokine levels in vivo. Subsequently, we examined the alterations in serum inflammatory cytokine levels among the five groups of mice. In this study, the ECP groups exhibited significant downregulation of the proinflammatory cytokines IL-17A (3.077 ± 0.401 pg/mL vs 0.238 ± 0.082 pg/mL, *P* < 0.0001) and IL-6 (53.47 ± 7.074 pg/mL vs 5.142 ± 1.779 pg/mL, *P* < 0.05) compared to the PBS group in the serum. Furthermore, interferon-γ and tumor necrosis factor levels were also lower in the ECP mice compared to the PBS group, although the trend did not reach statistical significance (Fig. 3f). Curiously, the levels of the anti-inflammatory cytokines IL-4 and IL-10, as well as the pro-inflammatory cytokine IL-2, were below the lower limit of detection in the assay.

### Depletion of monocytes during ECP treatment leads to ineffective therapy

After the depletion of monocytes by the EasySep^™^ Mouse CD11b Positive Selection Kit, the depletion percentage of monocytes was typically 84.0 ± 3.5% by flow cytometry (data were averaged over three experiments; Additional file 1: Figure S1). ECP-MD mice, in which monocytes were depleted during ECP treatment, did not exhibit improvements in body weight, arthritis score, or anti-CII levels (Fig. 1c–e). In fact, these conditions were either unchanged or worsened. Similarly, no amelioration of pathological symptoms was observed in CIA mice treated with ECP-MD (Fig. 2). Furthermore, ECP-MD treatment failed to induce an expansion of CD3 + CD4 + CD25 + Foxp3 + Tregs, and there was even an increased proportion of CD3 + CD4 + IL17A + Th17 cells compared to the PBS-treated group, resulting in a more severe imbalance of the Th17/Tregs ratio (Fig. 3c–e). Additionally, there were also no significant downregulations in the levels of anti-inflammatory cytokines (Fig. 3f). These findings suggest that the depletion of monocytes during ECP treatment leads to ineffective therapy and profitless immunomodulatory effects in CIA murine model.

### ECP in vitro trigger the differentiation of monocyte into tolerogenic dendritic cells

**Fig. 4:**
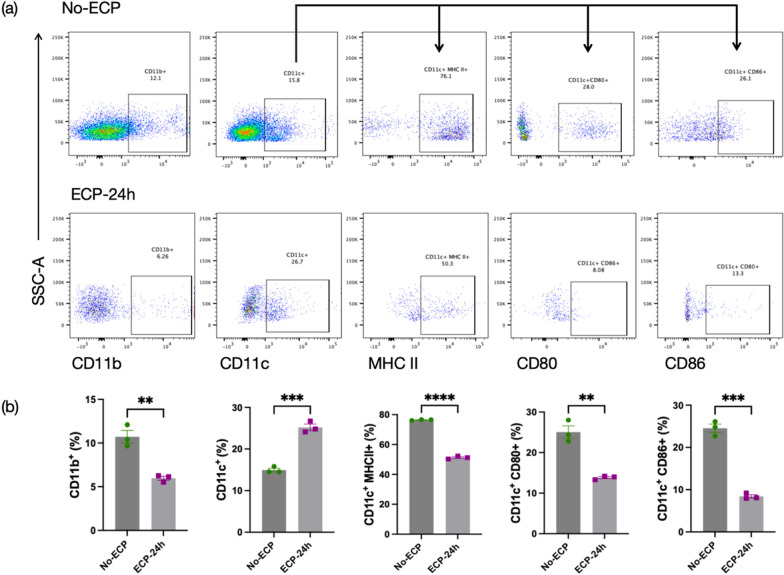
ECP in vitro trigger the differentiation of monocyte into tolerogenic dendritic cells. **a** The flow cytometry dot plots depict CD11b^+^ cells, CD11c^+^ cells, CD11c^+^MHC II^+^ cells, CD11c^+^CD80^+^ cells and CD11c^+^CD86^+^ cells in No-ECP and ECP-24 h group. **b** The proportions of CD11b + cells, CD11c + cells, CD11c + MHC II + cells, CD11c + CD80 + cells and CD11c + CD86 + cells in No-ECP and ECP-24 h group were determined three independent experiments and are represented as individual data points. Data are shown as mean ± SEM. *P ≤ 0.05, **P ≤ 0.01, ***P ≤ 0.001, ****P ≤ 0.0001.

To investigate the alterations in monocytes and dendritic cells following ECP in vitro, monocyte surface marker CD11b, dendritic cell surface marker CD11c, MHC II, co-stimulatory molecules (CD80 and CD86) were detected by flow cytometry (Fig. 4a). Compared with cells without ECP treatment, the proportion of CD11b + cells were significantly reduced (*P* < 0.01), the proportion of CD11c + cells were significantly elevated (*P* < 0.001). Additionally, the expression of MHC II (*P* < 0.0001), CD80 (*P* < 0.01), and CD86 (*P* < 0.001) was downregulated in CD11c + cells 24 h after ECP treatment (Fig. 4b). The observed decrease in CD80, CD86, and MHC II expression is characteristic of tolerogenic dendritic cells. The decrease of monocytes and the increase of tolerogenic dendritic cells suggest that ECP may induce monocytes to differentiate into tolerogenic dendritic cells.

## Discussion

Epidemiological surveys have shown that the global incidence of RA is 0.5 to 1% [25]. RA not only causes the decline of physical function, quality of life and social participation of patients, but also brings a huge economic burden to patients' families and society [26]. Considering its unfavorable overall prognosis, the principle of clinical treatment for RA is improve the disease, maintain low disease activity and preferably to relieve signs and symptoms (such as pain and joint swelling) [27]. To achieve this goal, patients need to undergo long-term drug administration. DMARDs are the most commonly used drugs in clinical practice, such as MTX, which is the anchor drug for RA treatment. However, the therapeutic response to DMARDs is often slow and incomplete, with still 50–60% of patients cannot achieve remission after treatment. Up to 60% of patients may require ≥ 3 treatment intensifications to achieve treatment goals [28]. A significant proportion of patients do not tolerate or respond to MTX monotherapy, necessitating the use of additional agents such as NSAIDs and glucocorticoids. However, these agents are associated with various side effects, such as bleeding and gastrointestinal ulcers caused by NSAIDs, and nausea, abdominal pain, ulcers, osteoporosis, and diabetes caused by glucocorticoids [29]. In addition to traditional treatments, biologic agents such as TNF-α inhibitors and IL-6 inhibitors represent a contemporary therapeutic frontier for RA treatment [30]. These biologics target specific inflammatory mediators that are central to RA pathophysiology. Despite their therapeutic efficacy, such agents are associated with considerable financial costs and an elevated risk profile, including the potential for opportunistic infections and an increased incidence of malignancies [31–33]. Moreover, RA patients frequently present with a spectrum of age-related comorbidities, necessitating polypharmacy that can lead to intricate drug-drug interactions, thereby complicating the management of RA. In comparison to commonly used clinical therapeutics, ECP demonstrates favorable safety profiles. As a treatment modality that induces immune tolerance rather than immunosuppression, ECP is not associated with opportunistic infections or cancer [34]. Adverse events are exceedingly rare; of the over half a million ECP treatments administered globally from 1987 to 2017, the reported incidence of adverse events was less than 0.003% [35]. The most common side effects included hypotension, transient anemia, transient febrile reactions, and headaches, all of which were sporadic and mild. In our investigation, based on our examination of symptoms, histology, immune cells, and cytokines in the CIA mouse model, we suggest that ECP may be a safer and effective treatment for RA. Furthermore, we have also explored the effect of ECP on the differentiation of monocytes into tolerogenic dendritic cells, offering a valuable reference point for subsequent comprehensive investigations into the underlying mechanisms of ECP.

We selected the CIA murine model in DBA/1J mice to assess the effectiveness and mechanism of action of ECP, primarily due to its ability to recapitulate the key clinical, histological, and immunological features observed in human RA. The two important characteristics of the CIA model are breach of tolerance and generation of auto antibodies towards self and collagen. Furthermore, among the antigen-defined models that rely on cartilage proteins, this particular model exhibits the shortest interval between immunization and the onset of disease manifestation [36, 37]. The model has been extensively utilized to evaluate the efficacy of MTX, a commonly prescribed drug for RA patients, in ameliorating clinical CIA scores [38]. Consequently, the CIA model serves as a valuable tool for evaluating the effectiveness of novel immunomodulatory therapies, with MTX commonly serving as a positive control for comparative analysis. Our findings indicate a notable exacerbation of clinical symptoms and pathological manifestations following collagen induction, thus validating the successful implementation of the CIA model in our study.

The ultimate goal of treatment is to achieve lasting relief of symptoms and synovitis and to prevent structural damage [39]. The joint tissues of both mice and humans are characterized by synovial hyperplasia, inflammation, and bone erosion [36, 40]. Besides, anti-type II collagen antibodies play an important role in the pathogenesis of CIA and are closely related to the severity of arthritis [41]. Therefore, it is primary to assess weight loss, joint swelling, levels of anti-type II collagen antibodies, and histopathological changes in animal models to evaluate the effectiveness of a novel treatment. Following treatment with ECP and MTX, we observed an effectively reduction in the severity of clinical symptoms and suppressed production of anti-CII antibodies in the CIA mice. Furthermore, pathological analysis and micro-CT imaging of CIA mouse confirm that ECP treatment attenuates bone and cartilage tissue damage in the ankle joints. These improvements demonstrated a consistent upward trend, indicating that ECP exhibits a therapeutic effect comparable to conventional therapy while offering a safer treatment option with minimal side effects in the context of RA.

RA is a chronic progressive autoimmune disease characterized by nonspecific inflammation of the peripheral joints [1]. While the precise mechanisms underlying the development of RA remain incompletely understood, current research indicates that the development and progression of the disease are characterized by an altered Treg response, which shifts towards a Th17 cells response [42]. Both Th17 cells and Tregs are regulated by a shared TGF-β signaling pathway, and proinflammatory signals in the RA dictate a reciprocal modulation of cell destiny [43]. For example, in the presence of interleukin IL-6 or IL-21, naïve CD4 + T cells differentiate into Th17 cells; however, in the absence of proinflammatory cytokines, TGF-β drives differentiation into Treg cells [42, 44]. Th17 cells predominantly promote inflammatory reactions through the secretion of IL-17, which in turn induces the production of pro-inflammatory cytokines (such as TNF-α, IL-6, interferon IFN-γ) that contribute to tissue invasion, irreversible damage to joint cartilage and bone, and the accelerated onset of autoimmune diseases [45, 46]. By contrast, Treg cells produce anti-inflammatory cytokines, suppress activity of a variety of immune cells, and thereby inhibit immune responses [47]. Thus, the dynamic interplay between these two cellular phenotypes is vital in modulating inflammatory and immune reactions [48]. Correspondingly, the upsurge in Th17 cells prevalence and pro-inflammatory cytokines was noted in our CIA mouse model, affirming that a skew towards Th17 dominance over Tregs correlates with aggravated RA manifestations.

ECP has exhibited therapeutic efficacy in the management of T cell-mediated diseases, particularly among the patients with GVHD, transplant rejections, and various autoimmune diseases. In murine models of GVHD, as well as in a majority of patients with GVHD, Tregs have been observed to proliferate in response to ECP [12, 49]. On the other hand, in some clinical trials focusing on systemic sclerosis undergoing ECP, in addition to the increase of Tregs in the peripheral blood of the patients, it was also observed that Th17 cells, which had a surge at the beginning of the disease, were decreased after ECP treatment and positively correlated with the improvement of skin symptoms [50, 51]. In some clinical study of ECP against rejection after solid organ transplantation, the regulation of Th17/Treg balance was also observed [52, 53]. Similarly, our research revealed that ECP precipitated the expansion of CD3 + CD4 + CD25 + Foxp3 + Tregs and reduced the populations of Th17 cells in the spleen, thereby facilitating immune modulation and alleviating RA symptoms. IL-6 is known to promote Th17 cells differentiation and concomitantly suppress Foxp3 via the phosphorylation and activation of STAT3 [44], while the presence of TNF-α amplifies this effect [54]. Our study shows that ECP treatment also reduced the proinflammatory cytokines (IL-17, IL-6, IFN-γ and TNF) and improved the inflammatory state in the CIA mice. These findings suggest that ECP effectively targets the balance between Th17 and Treg cells through its immunomodulatory actions.

Intriguingly, the previous study conducted by Céline Coppard et al. [55] reported that while ECP mitigated arthritis symptoms by reducing Th17 cell proportions, there were no significant alterations in the numbers of Tregs or in the levels of cytokines in CIA mice. This disparity may be ascribed to a shorter course of ECP sessions (three reinfusions) in their protocol, potentially insufficient for effectively rebalancing immune polarization and inflammation. In our current study, we observed that the therapeutic response in CIA mice treated with ECP was not discernible on the 2 day following the third infusion. Consequently, we augmented the number of treatment sessions to six reinfusions, administered every other day, leading to markedly enhanced therapeutic effects. In addition, diverging from previous methodologies wherein only spleen cells were procured from donor mice, our study utilized mononuclear cells from both the spleen and venous blood of donor CIA mice as the substrate for ECP treatment, which may also lead to better efficacy of mice and immune status in vivo. This approach more closely mirrors the clinical practice of peripheral blood collection and leukocyte layer isolation used in human ECP treatments.

Furthermore, we probed the crucial role of monocytes in the therapeutic effects of ECP. It has been suggested that the adherence to plastic surfaces during ECP, coupled with their interactions with platelets, may facilitate monocyte activation and their subsequent differentiation into dendritic cells [56–58]. In vitro investigations have demonstrated that ECP can induce the differentiation of monocytes into immature tolerogenic DCs [59], a process in which the activation of the glucocorticoid-induced leucine zipper (GILZ) gene by 8-MOP plays a decisive role in engendering tolerogenic dendritic cells [60], paving the way for the initiation of immune tolerance. However, knowledge of the monocytes in the context of ECP in vivo is particularly limited. Our in vivo findings indicate that monocyte depletion during ECP treatment results in a therapeutic impasse, with no improvement or even exacerbation of the condition in CIA mice. Therefore, it is postulated that the initiation of the therapeutic efficacy of ECP hinges upon monocytes. Our in vitro experiments suggest that ECP-treated monocytes may be induced to differentiate into tolerogenic dendritic cells by UVA and 8-MOP. After undergoing ECP in vitro, a notable reduction was observed in the proportion of CD11b + monocytes, while a significant increase was seen in the proportion of CD11c + dendritic cells. These CD11c + cells exhibited low expression levels of MHC II, as well as the costimulatory molecules CD80 and CD86, indicating the characteristics of tolerogenic dendritic cells. Functionally, tolerogenic dendritic cells have been observed to curtail effector T cell proliferation and foster Treg cell generation via direct cell-cell interactions and cytokine release in vivo, hence fostering immune tolerance [61]. Previous studies have indicated that tolerogenic dendritic cells possess the capacity to induce Treg cells and suppress Th17 cells in CIA mice [62], a trend corroborated in our own experiments. In conclusion, the cellular mechanism of ECP may involves the priming of monocytes to differentiate into tolerogenic dendritic cells through UVA and 8-MOP, with these cells subsequently exerting a therapeutic influence in CIA by reinstating Th17/Treg cells balance and immune tolerance. However, this path of action is blocked when monocytes are selectively depleted in the experimental setting, resulting in treatment ineffectiveness. Additionally, there are two ECP techniques in clinical use: the “inline procedure”, which combines cell collection, 8-MOP/UVA treatment, and reinfusion in a closed system; and the “offline procedure”, which involves separating and illuminating cells in separate devices [63]. The ECP described in this murine model more closely resembles the clinical offline procedure, which offers higher cell yields and greater opportunities for cell manipulation [64]. Therefore, additional clinical trials and relevant mechanism studies should be conducted further to support our findings.

## Conclusion

This study confirmed the therapeutic effects and preliminary mechanisms of ECP in the CIA murine model by the evaluation of RA clinical symptoms, the analysis of histological, the assay of Th17/Treg ratio and the cytokines of their spleen. ECP therapy effectively controls arthritis in CIA mice, indicating its potential as a therapeutic approach for RA. Notably, we emphasize the indispensable role of monocytes derived from CIA mice in facilitating the effectiveness of ECP treatment. The depletion of monocytes during ECP treatment renders the therapy ineffective, with the conditions of CIA mice either remaining unchanged or deteriorating. ECP may alleviate RA by promoting the differentiation of monocytes into tolerogenic dendritic cells, which in turn modulate the Th17/Treg balance and foster immune tolerance in vivo. These results are significant as they propose a possible therapeutic option and provide a reference direction for further in-depth investigation into the mechanism of ECP.

### Supplementary Information

**Additional file 1: Figure S1.** The representative dot plot of flow cytometry analysis of mononuclear cells suspension before and after depletion of CD11b+ monocytes by the EasySep^™^ Mouse CD11b Positive Selection Kit.

## Data Availability

Reasonable requests for data will be made available for review.

## Abbreviations

RA: Rheumatoid arthritis
ECP: Extracorporeal photopheresis
CIA: Collagen-induced arthritis
Foxp3: Forkhead box p3
Tregs: Regulatory T cells
Th: Helper T
8-MOP: 8-Methoxypsoralen
GVHD: Graft-versus-host disease
CFA: Complete freund’s adjuvant
IFA: Incomplete freund’s adjuvant
MTX: Methotrexate
