# Supplementary material for: Extracorporeal photopheresis reduces inflammation and joint damage in a rheumatoid arthritis murine model
Source: J Transl Med. 2024 Mar 25;22:305. doi: 10.1186/s12967-024-05105-x (PMC10962138; doi:10.1186/s12967-024-05105-x)
Supplement: Supplementary file 1 — Additional file 1: Figure S1. The representative dot plot of flow cytometry analysis of mononuclear cells suspension before and after depletion of CD11b+ monocytes by the EasySep™ Mouse CD11b Positive Selection Kit. [file 12967_2024_5105_MOESM1_ESM.pdf]

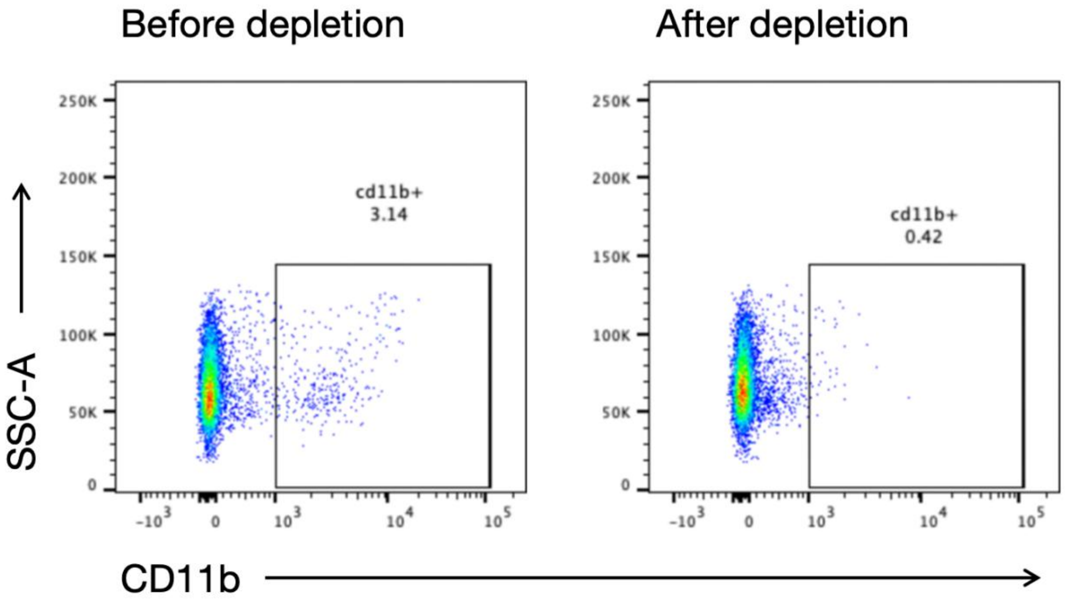

Figure S1

The representative dot plot of flow cytometry analysis of mononuclear cells suspension before and after depletion of CD11b+ monocytes by the EasySep™ Mouse CD11b Positive Selection Kit.
